# Breastfeeding peer support by telephone in the RUBY randomised controlled trial: A qualitative exploration of volunteers’ experiences

**DOI:** 10.1371/journal.pone.0237190

**Published:** 2020-08-06

**Authors:** Heather A. Grimes, Della A. Forster, Touran Shafiei, Lisa H. Amir, Fiona McLardie-Hore, Helen L. McLachlan

**Affiliations:** 1 Judith Lumley Centre, La Trobe University, Bundoora, Victoria, Australia; 2 School of Nursing & Midwifery, College of Science and Engineering, La Trobe University, Bundoora, Victoria, Australia; 3 La Trobe Rural Health School, Bendigo, Victoria, Australia; 4 The Royal Women’s Hospital, Parkville, Victoria, Australia; National University of Singapore, Singapore, SINGAPORE

## Abstract

**Background:**

There is growing evidence that peer support programs may be effective in supporting breastfeeding mothers. A randomised controlled trial (RCT) (the RUBY study) that tested peer support in the Australian context found that infants of first-time mothers who received proactive telephone peer support were more likely to be receiving breastmilk at six months of age.

**Methods:**

This qualitative sub-study of the RUBY RCT explores the experiences and views of peer volunteers who delivered the intervention. Focus groups were conducted with 17 peers from the RUBY RCT between November 2015 and March 2016. All had provided peer support to at least one mother.

**Results:**

We found that volunteers identified strongly with the mothers’ need for support when establishing breastfeeding. Key components of the support were strengthening the mothers’ self-belief through affirmation and sharing experiential knowledge. Volunteers found the role rewarding and personally therapeutic although some women reported challenges initiating and maintaining contact with some mothers. Data were analysed using a hybrid approach to thematic analysis combining inductive and deductive techniques

**Conclusions:**

Breastfeeding peer support programs are reliant on recruitment of motivated volunteers who can provide empathetic mother-to-mother support. This study provides important information regarding volunteers’ experiences that may support the upscaling of breastfeeding peer support for new mothers.

**Trial registration:**

Australian New Zealand Clinical Trials Registry, ACTRN 12612001024831.

## Introduction

Women who breastfeed possess experiential and embodied knowledge that has the potential to benefit new breastfeeding mothers and extend the duration of breastfeeding [1]. Lack of support has been identified as a reason for early breastfeeding cessation [2] and in societies in which women are isolated from breastfeeding role models [2] formal breastfeeding peer support programs present opportunities to share this valuable resource [1].

A Cochrane review comparing interventions providing extra support for breastfeeding mothers reported that compared to professional support, support provided by non-professionals reduced the risk of mothers not exclusively breastfeeding their babies to six months age [3].

Breastfeeding peer support has the potential to address gaps in support that make women vulnerable to early cessation of breastfeeding [1, 3–7]. A systematic review of 17 randomised controlled trials (RCTs)–with a meta-regression of 15 –found peer support was more effective in reducing the risk of non-exclusive breastfeeding in low and middle income countries compared to high income countries and when delivered at high intensity (≥ 5 contacts) compared to low intensity (< 5 contacts) [7]. The Ringing Up about Breastfeeding EarlY (RUBY) trial was an Australian RCT which evaluated the effectiveness of breastfeeding peer support by telephone and found that providing first time mothers with telephone-based support from a peer with at least six months personal breastfeeding experience was positively associated with higher breastfeeding maintenance at six months postpartum (75% giving breast milk in the intervention group Vs 69% in the control group; Adjusted risk ratio 1·10; 95% CI 1·02, 1·18) [8]. This is significant as previously, few interventions have been identified that increase breastfeeding duration in high income settings [7]. This article reports the findings from a sub-study of the RUBY RCT–the perspectives of the volunteers who provided the support.

Peer support roles are highly variable and embedded in programs with considerable heterogeneity [9]. Peers may be volunteers or renumerated and the experience they share with recipients may be as diverse as diabetes management, overcoming substance abuse, living with a mental illness or establishing breastfeeding. Method of delivery may be face to face, via web-based modalities or by telephone. A systematic review of seven RCTs assessing the evidence for telephone peer support interventions in health contexts, included three studies that reported on peer volunteers views and experiences [10]. The three studies [6, 11, 12] all reported qualitative data. The key themes emerging from the review were that peers needed to feel that they were helping recipients of support and while they valued sharing their personal experiences, they were sometimes confronted by the feelings that emerged including recollection of painful experiences and anxiety [10].

A qualitative meta-synthesis of 34 studies reporting the experiences of peers providing face-to-face support in the health context included five studies related to breastfeeding peer support [9]. Of those, one reported on postnatal telephone peer support [6] and the remainder reported interventions delivered in a variety of clinical and community settings [4, 13–15]. The study found that the peer support role enabled peers to reframe their identity through support relationships and secondly, the role constituted a ‘therapeutic use of self’ [9]. These constructs may evoke benefits or challenges for the peers. For example, sharing personal stories is potentially therapeutic for both recipient and peer. However, such benefits depend on achieving positive engagement and a sense of reciprocity within the relationship [9].

Providing peer support may confer benefits to peers including increased self-esteem and increased social-connection and potentially mitigates pre-existing feelings of isolation [6, 9, 16]. Frustration may arise however, when establishing and maintaining contact with recipients [17] and a perceived lack of engagement by recipients can be discouraging [9, 13]. Furthermore, it has been reported that volunteers in breastfeeding peer support programs may not participate for extended periods [18] which presents challenges in terms of ongoing recruitment and training. Defining boundaries between peer and professional breastfeeding support may require attention if the two are closely linked [15]. Findings from previous studies suggest that volunteers need ongoing support from supervisors to remain engaged and for quality assurance of programs [9, 10, 19].

Despite increased interest in peer support interventions, limited attention has been given to the experience of breastfeeding peer supporters. In addition, given the diversity of peer support programs, findings may not be generalisable across all contexts. In view of the success of the RUBY peer support model in extending the duration of breastfeeding, important questions arise in terms of what attracts volunteers to the role, the personal impact of providing support and their own needs for support. The aims of this study were to explore the experience of volunteers providing peer support to increase understanding of volunteers’ motivation for becoming peer supporters, and to describe their perceptions and experiences, in order to understand factors that may impact the duration of participation.

### The RUBY trial

The RUBY study was a two arm multi-site RCT that aimed to determine whether proactive peer support, provided in the postnatal period by telephone, increased the proportion of infants who were breastfed for at least six months. Further details of the RUBY trial can be found in the published protocol [20] and primary outcomes paper [8].

Between February 2013 and December 2015, 1152 primiparous women were recruited from three public hospitals in the state of Victoria, Australia during their postpartum hospital stay. Women were randomly allocated to usual care or telephone peer support in addition to usual care via a computerised system of randomisation designed and administered by an external party, accessed via the internet.

Volunteers who had breastfed for at least six months and were not considered professionals (and having previously had less than 8 hours of breastfeeding training) were recruited and trained to provide proactive telephone support to new mothers. Recruitment commenced in December 2012 and the final training session took place on May 2015. A total of 246 women completed the four-hour peer training session and 230 supported at least one mother. During training, an Australian Breastfeeding Association facilitator discussed normal infant behaviour, effective communication, existing resources, and sociodemographic factors that impact infant feeding.

Following allocation of a new mother, peers received the mother’s first name, phone number, baby’s date of birth and gender. Peers initiated calls to the mother at four to six days postpartum. Weekly calls were made for 12 weeks and contact tapered off to monthly calls until six months postpartum. Call frequency was adjusted if requested by the mother. Peers offered support with breastfeeding and general adjustment to parenthood, and directed women to existing local services as required [20].

## Methods

### Study design

We used a qualitative descriptive design [21] informed by the functional theory of volunteerism which provided a conceptual framework for interpreting data related to the motivation to volunteer [22]. Volunteering may be motivated by a desire to fulfil six functions which include a desire to express important personal *values*, to seek experiences to enhance skills or *understanding*, to form social connections or to enhance *career* prospects. Volunteering may also serve a *protective* function that offers a distraction from personal problems and finally, volunteer roles may serve an *enhancement* function that leads to a more positive self-appraisal [22].

### Data collection

All volunteers who provided support to at least one mother (n = 230) were invited by email to participate in a focus group between October 2015 and March 2016. A focus group guide was specifically developed to explore the issues thought to impact the volunteers and to elucidate their personal experience of providing support. Issues identified from relevant research literature, including the functional approach to volunteering, and collaborative discussion between the researchers informed the final interview guide [22]. Broadly, it explored (i) the reasons women chose to be a peer supporter; (ii) the type of support they provided; and (iii) their overall experience of volunteering.

Following each focus group, preliminary data analysis was undertaken, and the interview guide was reviewed in light of emerging themes. Two additional questions related to the volunteers’ experience of cultural diversity and its impact on the relationship they had with the mothers, as well as issues that arose related to establishing peer support boundaries were added after the first focus group.

Two researchers from the RUBY research team attended each focus group. Data collected were entered into a password protected Access database and hardcopies stored securely. The first author (HAG) transcribed and de-identified the recordings and the final transcripts were read independently by two researchers (HAG, HLM) and checked against the audio recordings.

### Data analysis

Data were analysed using a hybrid approach to thematic analysis that combined inductive and deductive techniques [23]. A coding schema was developed, *a priori* that aligned with the aims of the study and this directed initial categorisation of data. The categories included the motivations to volunteer, the nature of the support, and negative and positive aspects of providing support.

### Rigour

Methodological rigour was addressed using several strategies. The focus groups were facilitated by a chief investigator on the RUBY RCT who was experienced in qualitative methods. An associate researcher wrote field notes, and these were discussed with the facilitator immediately afterwards to check for consistency and interpretation of events. The transcripts were de-identified for analysis and individual participants were identified with the focus group and participant number (e.g. FG1, participant 3). This method identifies all quotes extracted verbatim from the data that were used to illustrate themes in this manuscript. The first author examined the transcripts line by line, and highlighted text related to the predetermined categories and new categories that emerged. The text was then re-examined and coded. HLM reviewed the codes independently and after discussion with HAG, codes were further refined and emerging themes identified. This was an iterative process. All authors participated in a final discussion and checking of themes to ensure they represented plausible findings from the original transcripts. An audit trail was maintained for each successive iteration of analysis.

### Ethics

Ethics approval for the RUBY RCT was obtained from the following Human Research Ethics Committees (reference number in brackets): Royal Women’s Hospital (12/25); La Trobe University (12–082); Monash Health (12251B); and Western Health (12/WH/107). Eligible women were recruited by research midwives during their postnatal hospital stay at one of the trial sites. Those who agreed to participate provided written consent prior to randomisation.

Volunteers who provided peer support to at least one woman and who responded to our invitation to participate in a focus group, were informed that participation was voluntary and were sent a participant information pack and consent form to read prior to attending. Facilitators collected the signed consent forms prior to commencing each focus group.

## Results

Four focus groups were conducted between November 2015 and March 2016, each lasting approximately 60 minutes. Data saturation and the study objectives were met following the fourth focus group when no new themes emerged [24].

### Participants

Of the 230 email invitations to participate, 34 peers responded and 17 participated. The main reasons given for not participating were return to paid work and lack of childcare. The demographic characteristics of participants are presented in Table 1.

**Table 1:** Participant characteristics (n = 17).

Characteristic	Number (n = 17)
**Age, years**	
30–34	4
35–39	9
40+	4
Mean years, (SD)	37.1 (4.2)
**Country of birth**	
Australia	12
England	1
South Korea	1
United States of America	1
New Zealand	1
Argentina	1
**English first language (n = 16)**	14
**Partnered**	16
**Income** (**n = 16)**	
$350 - $649 per week ($18,200 - $33,799 per year)	1
$650–$999 per week ($33,800–$51,999 per year)	1
$1400–$1999 per week ($72,800–$103,999 per year)	7
More than $2000 per week ($104,000 or more per year)	7
**Education**	
Completed a Degree or higher	13
Completed Diploma or certificate	3
Completed secondary school to Year 12 (or equivalent)	1
**Current employment**	
Employed full-time	2
Employed part-time	6
Maternity leave	4
Home duties	5
**Usual occupation**	
Education/ teaching	4
Health professional	3
Professional—other	3
Administration and/or management	3
‘Stay at home’ mother	2
Other	2
**Average number of children**	2 children
**Average age youngest child** (months) (range 4–48)	18 (SD 12)

Focus group participants were allocated a total of 66 mothers for peer support in the RUBY study, with an average of four mothers each (range 1–10). Their experiences ranged from never establishing contact with the mother (8/66, 12%) through to providing six months of support (28/66, 42%).

The focus of this study was the participants’ motivations to volunteer, the nature of the support they provided, and the positive and negative aspects of the role. Several themes were identified under each area of exploration. Key themes that emerged from the data are summarised in Table 2.

**Table 2:** Themes and subthemes.

Area explored	Themes	Sub-themes
**Motivation to participate in the program**	Women need breastfeeding support	*Empathy with mothers' personal journey;*
		*Volunteers’ own experience of breastfeeding support;*
		*Breastfeeding can be isolating*
		*Inconsistent or lack of breastfeeding support*
	Giving back	*Gratitude for support received when breastfeeding;*
		*‘Paying forward’*
	Flexibility of the role	*The role wouldn’t be too demanding*.
		*A good fit with other commitments*
**Type of support provided**	Building trust/ rapport	*Developing a relationship over time*
		*Developing rapport*
		*Listening to their story*
	Providing affirmation	*Affirming normal infant behaviour*
		*Promoting realistic expectations*
		*Buffering against stress*
	Providing information	*Sharing the experience of motherhood;*
		*Sharing information*
**Personal impact of providing support**	Personal benefits for the volunteers	*A therapeutic experience;*
		*Boosted my self-esteem;*
		*It feels good to help others*
	Contact challenges	*Anxiety about making the calls*
		*Frustration with unanswered calls;*
		*Disappointment when women didn’t engage with support*
	Cultural and linguistic challenges	*Language barriers*
		*Anxiety about lack of specific cultural knowledge*
		Opportunities to learn from CALD* *mothers*

* Women from culturally and linguistically diverse backgrounds.

### Motivation to participate in the program

In relation to volunteers’ motivation to become peer supporters, the main themes to emerge were ‘*new mothers need breastfeeding support’*, *‘giving back’*, and *‘flexibility of the role’*.

#### ‘New mothers need breastfeeding support’

A strong theme was the volunteers’ insight and understanding that new mothers need breastfeeding support. All volunteers referred to their own breastfeeding experience and the adequacy of the support they received. They highlighted the importance of support to overcoming challenges and described how feelings of loneliness and isolation exacerbated difficulties. They talked about the way in which new mothers may mask their struggles during this period in the face of ‘*so much pressure to look like you’re actually managing it when most people aren’t*.*’* (FG3, participant 1). One mother recalled that she ‘*didn’t really have support when (she) first had the baby and was feeling very isolated’* (FG1, participant 1) and another observed that *‘It’s quite lonely as a parent… We have probably all experienced that*. *And just to be able to have that connection with another mum is helpful’*. (FG1, participant 2).

Many recounted that they did not know who to trust for support or correct advice, and they valued support from other breastfeeding mothers. Family members may not have breastfed or could not recall the challenges. One volunteer commented that she ‘*had family support and all that*, *but they didn’t understand the difficulties’* (FG2, participant 1). Some volunteers received care from health professionals who they perceived lacked training in breastfeeding support. Peer support was viewed as a means of filling in information gaps:

‘*I felt really hesitant saying “Go to your GP [general practitioner]” because they… are highly unlikely to know anything about breastfeeding’*. *(FG3*, *participant 1)*

A key motivation for women to volunteer related to a deep, personal, empathetic awareness that women need support to breastfeed and that for some women, this may be lacking.

#### ‘Giving back’

Volunteers expressed gratitude for the positive support they received when establishing breastfeeding, and saw this as something valuable to share with others. Volunteers talked about ‘*looking for some way of giving back’* the support they had received. When volunteers could not directly reciprocate support received, they described ‘paying forward’ the support to others:

‘*It’s nothing out of my time*, *just looking out for someone else*, *and I think of that person that helped me*. *I just thought ‘…I can’t pay her back*, *but I can help someone else*. *Pay it forward*.*’* (FG1, participant 2)

#### ‘Flexibility of the role’

For many women being a telephone peer supporter enabled them to support mothers without interfering with their own family responsibilities or necessitating extensive training. The majority of mothers commenced volunteering when they were on maternity leave from paid employment, and the flexibility of the role was something they could fit in around their family commitments:

‘*I just thought it was a good thing to do and it wasn’t too time consuming in terms of I didn’t have to go anywhere*. *I had a training session and I could do it on the phone*.*’ (*FG1, participant 2)

Thus the nature of the role enabled volunteers to support mothers whilst being compatible with their own/personal commitments.

### Type of support provided

Volunteers were asked about the support they provided to the mothers. Key themes to emerge were ‘*building trust/rapport’*, ‘*providing reassurance and/or affirmation*’, ‘*providing information*’ and ‘*providing more than breastfeeding support*’.

#### ‘Building trust/ rapport’

The RUBY volunteers proactively telephoned mothers for up to six months after their baby was born. The initial calls were mostly an introduction and initiating the relationship:

‘*Making friends with the mums and winning their trust initially*. *Like “Congratulations*, *how’s it going”…kind of establishing that rapport…Trying to make that first initial contact…was the most tricky thing*. *Once that happened and I spoke to the woman*, *it was like holding her hand through the process’*. *(FG3*, *participant 2)*

Providing ongoing support to the same mother helped to build a relationship and increased the level of trust as they got to know each other. Fundamental to developing the trust required to build the relationship was actively listening to the mothers and hearing their story:

‘*You do need to have the conversation without distraction so the person on the other end of the phone feels like they’re being listened to and they are being heard*.*’* (FG4, participant 1)

The relationships varied in intensity from brief exchanges, to friendships that extended beyond the period of support. Some volunteers were sad when the period of support ended and would have liked to maintain the relationship:

‘*The mums who go the whole distance [six months]*, *you generally have a lovely relationship by the end*. *It’s almost a bit sad saying “Well this is my last phone call” and it seems a bit strange*.*’*(FG2, participant 2)

#### ‘Providing affirmation’

Through personal experience, volunteers were aware that new mothers may encounter stressors during the transition to motherhood, and that their ability to cope can be hampered by exhaustion and managing their own physical recovery. Volunteers were unable to directly alter the mother’s circumstances, but could reappraise the situation and in some cases assist in ‘…*giving them a different perspective from what they had’* (FG3, participant 3) and reassurance when things were ‘normal’:

‘*You just want someone to listen to you*, *someone to say*, *“This is absolutely normal” and you know*, *“You are doing a good job”*.*’* (FG1, participant 1)

The volunteers recognised that they could potentially undermine a mother’s confidence and felt it was important to reassure the mother that she was doing a good job:

‘*If your mum is already questioning everything she’s doing*, *you don’t want to seem like you know it all*, *and “this is how I did it and this is how it has to be”*.*’* (FG1, participant 3)

Several volunteers identified that mothers were vulnerable to criticism from friends and families, who did not always support breastfeeding. This could be expressed by negative comments or by uninformed opinions. Volunteers viewed peer support as a buffer against some of the stress caused by struggling to breastfeed within an unsupportive social environment. One volunteer described how *‘You’re basically coming in and being a little voice in their home that they can talk to*. *They can admit things to you that they can’t admit to a family who’s judging and being negative about what they are doing*.*’* (FG1, participant 1)

#### ‘Providing information’

Through their own breastfeeding experiences and those shared amongst their social network, volunteers possessed embodied and experiential knowledge that they could share with mothers. This ranged from giving specific advice and assisting with problem solving, to providing information about resources, or suggesting referrals that may be useful to the mother:

‘*Just having the experience and advice that I’d had and being able to translate that to real life support in a non-judgemental and non-directive manner*, *was nicely rewarding for me and I think for them as well*.*’* (FG1, participant 3)

The training session included advice about the boundary between peer and professional support. Focus group participants stated the type of support wanted by some of the mothers was not necessarily ‘professional’ support but support from another mother who had similar experiences: ‘*they…didn’t want professional help*, *they wanted to talk to me and get my experience*.*’* (FG2, participant 3)

### Personal impact of providing peer support

We asked volunteers to describe their experience of volunteering and any personal impacts. The main themes derived from the responses were ‘*personal benefits for the volunteers’*, ‘*contact challenges*’ and ‘*cultural and linguistic challenges*’.

#### Personal benefits for the volunteers

The volunteers identified a number of personal benefits that they had derived from volunteering in the program. One volunteer described the experience as being therapeutic in that it helped her to come to terms with her own negative experiences when establishing breastfeeding. The role helped them to reflect on their own experiences and in some cases, gain perspective on what they had been through:

‘*A lot of mums have trouble breastfeeding and … there can be a lot of mental damage done*. *I think doing this helps us reconcile some of that stuff ourselves…that what happened to me is in the past*. *Maybe if I help and listen to another mum*, *I’m perhaps giving her what I maybe didn’t get*, *or would have wanted more of*.*’* (FG1, participant 2)

Some women enjoyed the feeling of participating in something they considered worthwhile beyond their current role as a parent, reducing feelings of isolation, which were still quite real for some volunteers. Overall volunteers described increased feelings of self-worth gained from providing peer support:

‘*I’ve really enjoyed it*. *You know those days when your children are not the angels that you’d like them to be and you’re just doing boring thing after boring thing*? *It’s so nice to have a phone call with somebody you have actually helped*, *done something that was useful for somebody*, *because I’m a full-time mum at the moment*, *and my self-esteem is just a little low*.*’* (FG3, participant 1)

There was a strong sense of satisfaction derived from helping another mother, especially if the outcome was positive:

‘*I’ve gotten a lot out of it because I’ve had some really good experiences with mums continuing breastfeeding so I feel like my time has been valued*, *you know putting in*, *and they’ve gotten something out of it*.*’* (FG1, participant 1)

#### Contact challenges

Making the first phone contact was sometimes a stressful and/or exciting time for some peers. Volunteers were passionate and committed to providing support, but some described feeling apprehensive making the first call. They were unsure of the response they would get from the mother, or whether they would be able to provide the required support. This anxiety usually soon subsided with subsequent calls:

‘*I just didn’t know what sort of response I was going to get from the other end of the phone with the first*. *Not necessarily so much with the consequent mothers*, *but certainly with the first*.*’*(FG4, participant 1)

Some volunteers were personally disappointed if the mothers did not respond to the first or subsequent calls and became concerned about what was happening with the mother:

‘*I really worried about the women I couldn’t support*, *especially the first one*, *it was my first time doing it … and I know it’s ridiculous*, *but I felt anxious*, *“Oh God*, *has something really bad happened*?*”* (FG3, participant 2)

When the mother ended the period of support early or didn’t respond to calls, some of the peers reflected on it in a personal way. Strategies used for dealing with these situations included reframing the experience and acknowledging the mothers’ decisions about participation in the program:

‘*The first mum I did really take it personally*. *Like “What have I done*?*” But then I thought about it and like none of my friends breastfed their kids past 2 weeks*. *I’ve helped these mums like to at least a month*. *I just turned it around*.’(FG2, participant 1)

#### Cultural and linguistic challenges

Volunteers reported challenges when supporting women from culturally and linguistically diverse (CALD) backgrounds. The mothers were screened for English proficiency, however some volunteers had difficulty in understanding mothers with very strong accents. The lack of visual cues available during phone contact contributed to the problem:

‘*Her accent*, *in person would have been perfectly fine*, *but over the phone it was difficult*. *We managed to talk*, *but it was too hard to have a full conversation*.*’* (FG2, participant 1)

Lack of knowledge about the diversity in cultural practices in relation to infant feeding and broader postpartum practices caused some anxiety amongst peers. They were concerned about causing offence by saying the wrong thing, and expressed apprehension about providing advice that may conflict with that given by respected family members:

‘*I know one of the mother’s mother was constantly telling her to formula feed*. *And I know that was a cultural thing*. *It’s just the way it is done*, *they always formula fed … It was very hard to get past that*.*’* (FG3, participant 3)

One volunteer questioned whether coming from different cultural backgrounds limited her capacity to be an empathetic peer to the CALD mothers supported:

‘*Can you truly be a peer if you come from a different cultural background*? *I think the strength [of peer support] is that you can empathise with all the stuff that goes on behind breastfeeding and having a baby… If you’re coming from a very different cultural perspective*, *can you truly be on exactly the same level playing field*?*’* (FG3, participant 2)

This generated discussion and another volunteer concluded that although sociodemographic differences existed between her and her peer mother, the shared experience of motherhood gave them common ground:

‘*I’m a lot older than most of the women I spoke to and I also suspect I’ve got a lot more education than all of them too*, *and … you’re constantly negotiating those differences*. *I kept trying to come back to “What does it feel like to be a mum for the first time*?*” Because in that you really are stripped of a lot of your… worldly signifiers*.*’* (FG3, participant 1)

## Discussion

The RUBY trial demonstrated that proactive peer support by telephone in the postnatal setting increased the number of infants receiving breastmilk at six months of age. Given the significance of the findings it is important to explore the experiences of the peer volunteers. This qualitative study took place in the final months of the RCT, before the primary study outcome was known. A qualitative component was included to enhance understanding of how the intervention was implemented and to explore factors that might impact scaling up of the program [25]. This study sheds light on the acceptability of the intervention to those providing support and findings suggest that peers found the role rewarding, and experienced mutual benefit from sharing their breastfeeding experiences. They reported challenges in initiating and maintaining contact, and communication difficulties with culturally and linguistically diverse women and this study highlights the importance of providing ongoing support to peer support volunteers.

The functional theory of volunteerism provided a conceptual framework to guide the interpretation of the peers’ motivation to volunteer [22]. A number of motives were identified although a strong sense of breastfeeding advocacy and concern for the plight of new mothers emerged as most important. This is consistent with the values function described in the volunteer functional inventory [22]. Participants viewed breastfeeding as a positive health behaviour that mattered to the well-being of mothers and infants.

In this study, volunteers described how the role was personally therapeutic and, in some instances, resolved negative feelings related to their own experiences of breastfeeding. These findings are supported by several studies that report that sharing challenging experiences in the course of peer relationships may confer mutual benefits by enabling validation and reframing of personal stories and a subsequent sense of closure [9, 16, 17]. Volunteering has been widely reported to increase self-esteem, self-efficacy and social connectedness [15] and for mothers with childcare responsibilities, social interaction may combat the isolation associated with early motherhood [4]. A peer support project for mothers of preterm infants found that issues could arise if peers have unresolved emotions related to their own experiences and a 12-month period between the peers’ experience and undertaking a peer support role has been suggested [26]. Those issues where not apparent in this breastfeeding study although all volunteers has been breastfeeding for at least six months.

Peers viewed the support they provided as unique and grounded in their direct experience of breastfeeding rather than a substitute for professional support. Whilst health professionals are important providers of breastfeeding support and information, the volunteers’ personal experiences led them to conclude that this was not always reliable. Studies have reported substantial gaps in knowledge and skills related to breastfeeding amongst health professionals [27] and this is may have a negative impact on women trying to overcome breastfeeding challenges. Women have also reported feeling less rushed when receiving support from peers compared to health professionals [28]. This study supports the view that peer support is unique and although some elements overlap with health professional support, it fulfils different needs for breastfeeding mothers.

Volunteers reported that ongoing contact with the same mother enabled trust and rapport to develop. Engaging recipients during initial contacts and developing ongoing rapport is crucial to sustaining relationships [6] and achieving ‘authentic presence’ with mothers [28]. When peers perceive that calls are appreciated, there are compounded benefits in that they may give more attention to relationships that are valued by the recipient [29]. Programs enabling ongoing contact between individual peers and recipients foster relationships that may be more satisfying for peers.

In instances where peers and mothers were culturally diverse, we found that if English skills were sufficient for adequate telephone communication, a successful peer relationship could be established. It is not surprising, however, that this study confirms previous findings that language barriers can have a negative impact on the provision of peer support [6, 13, 17]. The extent to which the peer relationship can successfully navigate cultural differences per se and find ‘common ground’ in the shared experience of motherhood is less clear. Greenwood and Habbi [30] suggest that in a crisis, people learn effective coping strategies from those who have been in comparable situations. Focusing on the mutual experience of breastfeeding during interactions as a means of identifying ‘common ground’ may assist in sustaining the relationship. Sociodemographic diversity can also be framed as an opportunity to share valuable and mutually beneficial cultural and language insights [31].

Peer relationships are not without stressors and at times volunteers had concerns about aspects of the role. As found in previous studies, pragmatic challenges in contacting recipients and uncertainty that arises if contact isn’t made, can impact on peer morale [13]. Providing support by telephone avoided issues associated with face to face contacts such as travel pressures [32]. Regular contact from a peer support coordinator promotes ongoing peer engagement and provides guidance and support of peers who face challenges [18]. In addition, ongoing contact supports quality assurance of peer programs by promoting adherence to intervention guidelines [9, 19] and leads to decreased attrition rates [26].

### Strengths and limitations

There is limited in-depth, qualitative research focused on the experience of volunteers who provide breastfeeding peer support. Focus groups provided an opportunity to elicit more detail about volunteers’ personal experiences of providing peer support than would have been possible by survey alone. Volunteers with diverse peer support experiences in terms of the duration of support and number of women supported provided a range of views. Those who attended may have been more satisfied with their experience or more motivated about peer support than those who did not participate. Consequently, the views expressed may not be those of all volunteers.

## Conclusion

The volunteers in this study demonstrated an empathetic understanding and commitment to help breastfeeding mothers, gained through their own personal experience. Providing peer support was largely a positive experience that provided psychosocial benefits to volunteers. Challenges related to difficulties initiating and maintaining contact, and language barriers. The findings of this study support previous research that highlights the need for volunteer peer supporters to receive regular support from program coordinators to help them navigate challenges that may arise.

Despite volunteers describing the role in mostly positive terms, further studies could help to identify modifiable factors that extend the duration of volunteers’ participation.

The findings suggest the peer support role offers mothers the opportunity to share valuable breastfeeding knowledge and encouragement to new mothers within a relationship that may be mutually beneficial. In addition, by highlighting aspects of the volunteers’ experience that may support recruitment and management of peers, these findings support the sustainability of the peer support model offered within a study context. The RUBY trial demonstrated that volunteer peer support is one of few strategies to increase breastfeeding duration. In view of this, and the acceptability of the volunteer peer support role reported in this study, research translation activities within community settings are warranted.

## Supporting information

S1 File RUBY volunteer focus group questions. (DOCX)
