# Supplementary material for: Breastfeeding peer support by telephone in the RUBY randomised controlled trial: A qualitative exploration of volunteers’ experiences
Source: PLoS One. 2020 Aug 6;15(8):e0237190. doi: 10.1371/journal.pone.0237190 (PMC7410279; doi:10.1371/journal.pone.0237190)
Supplement: S1 File — (DOCX) [file pone.0237190.s001.docx]

**Focus group aims:**

- To find out what factors led to volunteers commence/continue to participate
- To generate discussion amongst volunteers about their preparation for the role of RUBY volunteer
- To generate discussion about the impact providing telephone peer support had on the volunteers

|  | **Theme** | **Question** | **Prompts** |
| --- | --- | --- | --- |
| **Antecedents**  *Motivations, expectations,*  *planned duration* | Motivation to volunteer | **Can you recall what prompted you to volunteer for RUBY?** | Support BF, volunteering, able to do from home |
|  | Expectations of role | **Was the role what you expected?** | Meeting vols/ personal expectations.  Satisfaction/ dissatisfaction |
|  | Duration of volunteering | **Can you tell me some of the reasons you continued to support mothers for as long as you did?** | Sense of commitment…  Promotion of BF…  See them through’…  Satisfying aspects of role |
| **Experiences**  *Focus on what the role entailed, experience of training & support* | Role of the volunteer | **Can you tell us about the support you provided to the mothers you were allocated** | Social contact for mothers, providing information & encouragement. Following the call schedule |
|  | Preparation for role of providing emotional/ appraisal and informational support | **Do you have any comments about the training session you attended before you started in the role?** | Did you feel prepared for the role when you started? The first call..  Content and length of session, follow-up sessions, resources such as handbook |
| **Consequences**  *Future intentions, duration of participation, well-being of volunteer – positives & negatives* | Impact on you | **Can you tell me about how the experience of volunteering was for you personally?** | Positive, negative aspects. Anxiety, guilt, feelings of intrusion, connection, prosocial activity.  What have been the benefits for you? What have been the costs? |
| **Concluding question** | Do any of you have anything at all that you would like to add? | | |
